# Supplementary material for: Walking around the Autonomous Province of Trento (Italy): An Ethnobotanical Investigation
Source: Plants (Basel). 2022 Aug 29;11(17):2246. doi: 10.3390/plants11172246 (PMC9460053; doi:10.3390/plants11172246)
Supplement: Supplementary file 1 [file plants-11-02246-s001.zip › plants-1840749-supplementary.pdf]

Table S1: Plants cited by the informants exploited for liquor preparation

Scientific Name	Habitat	Plant part used	Balsamic period	Preparation	Pathology
Achillea erba-rotta All.	Grasslands	Aerial parts	July-August	Steeping in grappa	-
Aloysia citrodora **Palau**	Cultivated	Leaves	July	Steeping in grappa	-
Artemisia nitida Bertol.	Rock fissures - stony pastures	Whole plant	July	Spirit	Dyspepsia
Cornus mas L.	Woods - Hedges	Berries	September	Steeping in grappa	-
Galium odoratum L.	Woods	Whole plant without roots	Spring	Steeping in grappa	-
Juglans *sp.pl.*	Cultivated - Woods	Nuts	When nuts are green	Steeping in grappa	Dyspepsia
Nigritella nigra **(L.) Rchb.**	Grasslands	Flowers	Summer	Steeping in grappa	-
Pinus mugo Turra	Dwarf Pine scrub	Buds, green cones	Summer	Steeping in grappa<br>Spirit	-<br>-
Prunus spinosa L.	Woods - Hedges	Berries	Autumn	Spirit	Dysentery
Vaccinium myrtillus L.	Woods - Heaths	Fruits	Summer	Steeping in grappa	-

Table S2: Plants cited by the informants exploited for veterinary purposes

Scientific Name	Habitat	Plant part used	Balsamic period	Preparation	Pathology
***Elymus repens* (L.) Gould *Syn. Agropyrum repens* L**	Grasslands	Rhizomes	Summer	Raw	Diuretic
***Artemisia absinthium* L**	Meadows - Forest undergrowth	Aerial parts	Summer	Infusion	Cow dyspepsia
***Leontopodium alpinum* Cass**	Pastures	Flowers	Summer	Infusion	Cows dysentery
Malva sylvestris L.	Grasslands – waysides	Leaves e Flowers	Summer	Infusion	Cow dyspepsia
***Medicago sativa* L.**	Grasslands - Cultivated	Aerial parts	Summer	Feed	-

Table S3: Plants cited by the informants exploited for medicinal purposes

Scientific Name	Habitat	Plant part used	Balsamic period	Preparation	Pathology
Abies alba Mill.	Woods	Resin	-	Resin blisters	Muscle and joint pain
		Needles	-	Essential oil	Diseases of the respiratory system
Achillea millefolium L.	Meadows	Flowering tops	Summer	Infusion	Menstrual pain and oligomenorrhea
				Oleolite	Muscle and joint pain
***Elymus* repens (L.) Gould Syn.** *Agropyrum repens* L.	Grasslands	Rhizomes	Summer	Decoction	Urinary tract infections
Arctium lappa L.	Tall herb fringes	Fresh leaves	July	Skin applications	Bug bites
		Roots	Autumn	Decoction	Diuretic
Arnica montana L.	Grasslands	Flowers	July	Tincture or Oleolite	Muscle and joint pain, muscle strain
				Pack	Sprains
Artemisia absinthium L.	Meadows, roadsides	Branches	Summer	Raw branches	Dyspepsia and worms
				Pack with powdered branches	Sprainse rheumatism
		Leaves and Flowers	May - June	Infusion	Dyspepsia
Bryonia alba L.	Wood edge - hedges	Roots	Autumn	Tincture	Muscle and joint pain
Calendula officinalis L.	Cultivated	Flowers	June - July	Oleolite	Wounds, burns
***Capsella bursa-pastoris* L.**	Cultivated Roadsides - Meadows	Basal rosette	June - August	Infusion	Fevers, diarrhea, urogenital diseases
				Raw	
				Tincture	Rheumatism
Carum carvi L.	Pastures	Seeds	Summer	Infusion	Dyspepsia, bloating and a swollen belly

Scientific Name	Habitat	Plant part used	Balsamic period	Preparation	Pathology
***Senna alexandrina* Mill. Syn. *Cassia angustifolia* Vahl**	Cultivated	Leaves and fruits	Summer	Infusion	Constipation
***Cetraria islandica* L.**	Alpine and subalpine dwarf shrub formations	Whole plant	Summer	Syrup	Diseases of the respiratory system
Chelidonium majus L.	Waysides – Hedgerows – Woods edges	Latex	Summer	Direct application	Corns, calluses and warts
Cornus mas L.	Woods - Hedges	Berries	Septembe r	Gelatine	Dysentery
Crataegus monogyna Jacq.	Woods - Edges	Flowers	Spring	Infusion	Hypertension and arrhythmias
Cyclamen purpurascens Mill.	Woods	Bulb	July-August	Oleolite	Ear infection
Equisetum arvense L.	Cultivated fields	Buds	May - July	Infusion	Diseases of the urogenital system
Gentiana lutea L.	Pastures	Roots	Autumn	Decoction or Tincture	Dyspepsia
Hypericum perforatum L.	Pastures - Dry grasslands - Hedges	Flowers	July	Infusion<br>Oleolite<br>Infusion	Infections of the respiratory system<br>Diseases of the skin<br>Depression
Juniperus sp.pl.	Shrubs - Woods	Berries	Autumn	Infusion<br>Steeping in grappa	Dyspepsia and urinary tract infections<br>-
Larix sp.pl.	Woods	Resin<br>Fresh female flowers	Autumn<br>Summer	Ointment<br>Steeping in grappa	Cold, herpes, insect bites with abscesses and cuts on the fingerscon<br>-
***Leontopodium alpinum* Cass**	Pastures	Flowers	Summer	Extract	Skin aging
***Lycopodium clavatum* L.**	Pastures – Heaths - Woods	Flowers	Summer	Powdered flowers	Disease of the skin

Scientific Name	Habitat	Plant part used	Balsamic period	Preparation	Pathology
Malva sylvestris L.	Grasslands - waysides	Leaves and Flowers	May - June	Compress, Infusion Decoction	Muscle aches, oral and eye infections
		Fresh leaves	June - Septembe r	Infusion	Dyspepsia
Matricaria chamomilla L.	Weed vegetation	Flowers	July	Compress<br>Infusion	Conjunctivitis<br>Dyspepsia and muscle aches
Melissa officinalis L.	Ruderal vegetation - Cultivated	Leaves	June - July	Infusion	Insomnia and premenstrual syndrome
Nepeta cataria **L.**	Ruderal vegetation – Clearings (1000 m)	Leaves	Summer	Infusion	Muscle aches, Insomnia and premenstrual syndrome
Oxalis acetosella **L.**	Forest undergrow th	Flowers	Summer	Ingredient for food	Stomach Acidity and Dyspepsia
Papaver rhoeas **L.**	Weed vegetation - Waysides	Petals	July	Infusion	Insomnia and cough
Picea abies **(L.) H.Karst.**		Needles	-	Essential oil	Diseases of the respiratory system
Pinus cembra **L.**	Woods	Cones	Summer	Syrup	Diseases of the respiratory system
Pinus mugo Turra	Dwarf Pine scrub	Buds, green cones	Summer	Syrup	Diseases of the respiratory system
		Needles	Spring	Essential oil	
Plantago lanceolata **L.**	Meadows	Leaves	Spring and Summer	Infusion<br>Pounded leaves	Catarrh<br>Wounds and insect bite
Portulaca oleracea L.	Cultivated fields	Leaves and flowers	Late summer	Food<br>Infusion	-<br>Worm infection
Potentilla reptans **L.**	Meadows	Leaves and flowers	Spring and Summer	Infusion	Diarrhea and fever

Scientific Name	Habitat	Plant part used	Balsamic period	Preparation	Pathology
***Pulmonaria officinalis* L.**	Forest undergrowth	Leaves	Spring	Infusion	Cough
***Frangula alnus* Mill. *Syn. Rhamnus frangula* L.**	Hedges - Woods	Bark	Autumn	Infusion	Constipation
***Rhododendron ferrugineum* L.**	Shrubs - Woods	Galle	Autumn	Oleolite	Gout, joint pain
***Rhodiola rosea* L.**	Dry grasslands	Roots	Autumn	Infusion	Depression
Rosa canina L.	Wood margins - Hedges	Berries	Late October	Food<br>Infusion	Cough and sore throat
Sambucus nigra L.	Woods - Hedges	Flowers	Spring	Infusion	Cold
Satureja montana L.	Dry stony grasslands - Waysides	Leaves and flowers	Summer	Infusion	Digestive, reduces nervous gastric pains, bloating, and vomiting
***Hylotelephium telephium* (L.) Holub Syn. *Sedum telephium* L.**	Wood margins – Dry grasslands	Leaves	Spring and Summer	Raw leaves	Dermatitis, hemorrhoid abscesses, subside sores or ulcers, wound healing
Solanum nigrum L.	Cultivated fields – Ruderal vegetation	Berries	Summer		Warts and dermatitis
Solidago virgaurea L.	Grasslands - Woods	Flowering tops	July-August	Infusion	Cystitis
***Filipendula ulmaria* (L.) Maxim.**	Meadows (up to1500 m a.s.l.)	Flowers	Summer	Tincture	Muscle and joint pain
***Symphytum officinale* L.**	Forest undergrowth	Roots	Autumn	Oleolite	Muscle and joint pain, muscle strain
Taraxacum* F.H.Wigg. *sect. Taraxacum Syn. *Taraxacum officinale* **(L.) W.W.Weber ex F.H.Wigg.**	Meadows – Wet grasslands	Roots	Autunno	Decoction	Diuretic

Scientific Name	Habitat	Plant part used	Balsamic period	Preparation	Pathology
Tilia sp.pl.	Woods - Cultivated	Flowers	Late June	Infusion	Cough, cold, and tachycardia
	Woods - Cultivated	Flowers	Late June	Syrup	Insomnia
Thymus pulegioides **L. Syn.** ***Thymus serpyllum*** **L. pl.pl.**	Dry grasslands	Aerial parts	July	Infusion	Infections of the respiratory system
Tussilago farfara **L.**	Pioneer vegetation on bare soil	Flowers	February	Syrup	Diseases of the respiratory system
	Undergrow th	Leaves and flowers	Before flowers	Infusion	Diseases of the respiratory system
Urtica dioica **L.**	Wastelands – forest margins - Waysides	Young plants	Spring	Infusion	Idem
				Poultices	Skin inflammation and wounds
				Compress	Wounds
Vaccinium vitis-idaea **L.**	Woods – Heaths	Leaves	August	Infusion	Cystitis and colic
Verbascum thapsus **L.**	Waysides – Ruderal vegetation	Flowers	Summer	Infusion	Infections of the respiratory system
Viburnum lantana **L.**	Dry woods edges	Trichomes	-	Powder	Epistaxis
Viola odorata **L.**	Grasslands – Hedges - woods edges	Leaves and flowers	Spring and Summer	Infusion	Cough
